# Contact residue contributions to interaction energies between SARS-CoV-1 spike proteins and human ACE2 receptors

**DOI:** 10.1038/s41598-020-80942-6

**Published:** 2021-01-13

**Authors:** Jorge H. Rodriguez, Akshita Gupta

**Affiliations:** Computational Biomolecular Physics Group, Department of Physics and Astronomy, Purdue University, West Lafayette, IN 47907-2036 USA

**Keywords:** Biophysics, Molecular biophysics, Virology, SARS-CoV-2, SARS virus, Structural biology

## Abstract

Several viruses of the *corona* family interact, *via* their spike (S) proteins, with human cellular receptors. Spike proteins of SARS-CoV-1 and SARS-CoV-2 virions, being structurally related but not identical, mediate attachment to the human angiotensin-converting enzyme 2 (hACE2) receptor in similar but non-identical ways. Molecular-level understanding of interactions between spike proteins and hACE2 can aid strategies for blocking attachment of SARS-CoV-1, a potentially reemerging health threat, to human cells. We have identified dominant molecular-level interactions, some attractive and some repulsive, between the *receptor binding domain* of SARS-CoV-1 spike proteins (S-RBD) and hACE2. We performed fragment-based quantum-biochemical calculations which directly relate biomolecular structure to the hACE2...S-RBD interaction energy. Consistent with X-ray crystallography and cryo-EM, the interaction energy between hACE2 and S-RBD ($\approx -$26 kcal/mol) corresponds to a net intermolecular attraction which is significantly enhanced by inclusion of *dispersion* van der Waals forces. Protein fragments at the hACE2...S-RBD interface, that dominate host-virus attraction, have been identified together with their constituent amino acid residues. Two hACE2 fragments which include residues (GLU37, ASP38, TYR41, GLN42) and (GLU329, LYS353, GLY354), respectively, as well as three S-RBD fragments which include residues (TYR436), (ARG426) and (THR487, GLY488, TYR491), respectively, have been identified as primary attractors at the hACE2...S-RBD interface.

## Introduction

The severe acute respiratory syndrome coronavirus, SARS-CoV-1, represents a potentially reemerging and not fully understood health threat^1,2^ that originated in late 2002. While the threat from SARS-CoV-1 faded with the aid of effective health mitigation policies, other coronaviruses have recently emerged including the genetically related SARS-CoV-2^3–6^. Although related, SARS-CoV-1 and SARS-CoV-2 display important differences in their host-binding structures, namely the *receptor binding domains* (RBD) of their spike (S) proteins^7^. Both reasons, public health concerns and RBD structural variations, underscore the need to study molecular-level interactions of each particular coronavirus with host-cell receptors. Such studies can elucidate the physico-chemical origins and residue-level mechanisms of viral infection. This work presents a quantitative structure-based analysis of key interactions, largely responsible for an attractive host-virus binding energy, between SARS-CoV-1 and the human ACE2 receptor.

**Figure 1:**
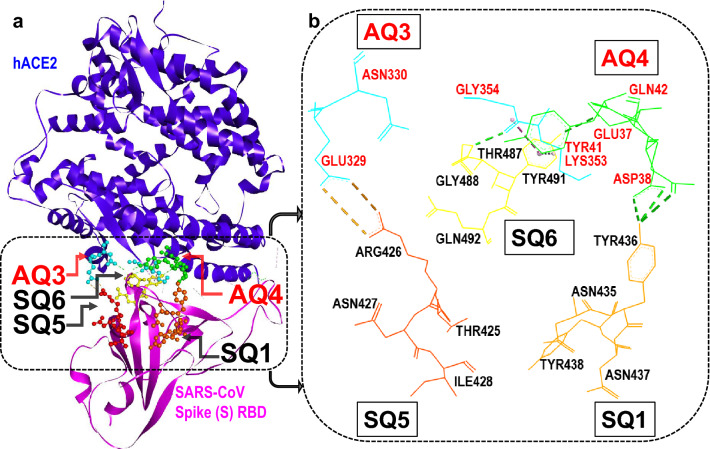
Identification of four-residue fragments (i.e. *quartets*) which produce attractive interaction energies between hACE2 and the SARS-CoV-1 S-RBD. (**a**) Structure of hACE2 receptor (Chain A) in complex with SARS-CoV-1 spike protein (Chain E)^3^. The key *quartets*, at the hACE2...S-RBD interface, promoting host-virus binding are shown in the dashed box. (**b**) Magnified view of the hACE2 (**AQ3**, **AQ4**) and S-RBD (**SQ1**, **SQ5**, **SQ6**) residue *quartets* which mostly contribute to the attractive hACE2...S-RBD interaction energy. Residues for each *quartet* are labelled.

Virions of the coronaviruses SARS-CoV-1 and SARS-CoV-2 have characteristic roughly-spherical shapes, on the order of 100 nm in diameter, with petal-shaped spikes which project outwards from their surfaces.^4^ Coronaviruses encode three types of surface proteins^5,8^, namely membrane (M), envelope (E) and, of particular importance, the so-called spike (S) which are positioned in their membrane envelopes. In addition another, nucleocapsid (N), structural protein is encoded. Spike proteins play crucial roles in a virion’s infection of host-cells, both by binding to their cellular receptors and, subsequently, by promoting fusion with their cellular membranes^4,9,10^. To interact with their host-cell receptors, spike proteins undergo conformational motions that either hide or expose their structural determinants of receptor binding which correspond to their *down* or *up* states, respectively^11^. Spike glycoproteins of coronaviruses play crucial roles in the initial stages of host-cell infection and are major targets for virus-neutralizing antibodies^12^. Thus, identifying and studying in a quantitative way the physico-chemical interactions between the *up*-state spike conformations, which are receptor accessible^11,13^, and their host receptors is of interest not only for elucidating the molecular-level origins of host-virus binding but also for developing therapeutic countermeasures.

Structurally, the spikes of coronaviruses are globular trimers, of about 150 $\AA$ in diameter, linked to the virion envelope by a narrow stalk. The spikes of SARS-CoV-1, in particular, are fairly massive ($\approx$ 500 kDa) in comparison to other type I viral spike proteins^9^. Spike proteins of both viruses, SARS-CoV-1 and SARS-CoV-2, contain two domains labelled S1 and S2. The S1 domain mediates initial virus binding to target cell receptors whereas S2 is involved in the fusion of virus and target cell membranes^1^. As shown in Fig. 1 spike (S) proteins interact with host receptors *via* their *receptor-binding domain* (RBD) which is herein referred to as S-RBD. A representative S-RBD which is closely related to that of SARS-CoV-1, namely that of SARS-CoV-2, has a molecular weight of $\approx$ 21 kDa and its prefusion cryo-EM structure has been recently reported^11^.

Coronavirus spike proteins, such as those from SARS-CoV-1, need to interact with receptors of their target cells to initiate infection. The human angiotensin-converting enzyme 2 (hACE2), attached to the outer surface of host-cells, has been identified as an efficient binder of the S1 domain of SARS-CoV-1 spike proteins^10,14^. The hACE2 motif was also identified as an entry receptor for the novel SARS-CoV-2 coronavirus S-protein^15–17^. Thus, blocking S-protein interaction with hACE2 or promoting hACE2 conformational changes that render it inefficient as a receptor are possible antiviral countermeasures. Conversely, identification of hACE2 receptors as viral entry points highlighted the role of spike protein RBDs as possible target epitopes of S1-protein-based vaccines^14^.

An important determinant of infectivity is the cognate interaction between viral attachment proteins and their host-cell receptors^18^. The structural basis for host receptor recognition has been reviewed for several coronaviruses and binding similarities as well as differences have been highlighted^19^. The main contact residues at the interface of the SARS-CoV-1 S-RBD with ACE2 receptors from several species, including human ACE2, have been structurally identified^3^. The crystallographic structure of the SARS-CoV-1 S-RBD, in complex with hACE2, was reported at 2.9 $\AA$ resolution^3^ and more recent Cryo-EM structures provide additional insight about prefusion to postfusion conformational changes^13,20^. Likewise, X-ray diffraction and Cryo-EM structures of the structurally related S-RBD of SARS-CoV-2^11^ and its complex with hACE2 receptors^21,22^ have been reported.

Recent molecular dynamics simulations have probed the interplay between spike proteins and hACE2 and presented comparisons between the binding mechanisms and/or affinities of SARS-CoV-1 and SARS-CoV-2^23–25^. Consistent with a variety of recent experiments^7,11,22,26^, molecular dynamics studies generally show an enhanced binding free energy for the S-RBM of SARS-CoV-2 relative to that of SARS-CoV-1. Structural, energetic and/or mechanistic roles of individual contact residues, including their hydrophobic or hydrogen bonding properties^24^, for each of these two S-RBDs have been discussed and comparisons presented with the goal of explaining the stronger binding free energy of the SARS-CoV-2 S-RBD^23,25^.

In this work we focus on the interaction energy of SARS-CoV-1 which, as defined by Eqs. (1-3), is a measure of the propensity of an S-RBD to attach itself to the hACE2 receptor. Techniques such as X-ray crystallography (XRC) and cryogenic electron microscopy (cryo-EM) can identify the contact residues at the hACE2...S-RBM interface. However, these techniques cannot unequivocally determine which S-RBM residue fragments are attractive or which are repulsive relative to hACE2. Likewise, XRC or cryo-EM cannot quantify partial hACE2...S-RBM interaction energies. By contrast, such information, helpful for antiviral or vaccine development, can be obtained *via* rigorous quantum-biochemical calculations as shown in the present study. Quantum-biochemical calculations^27^ can, to a large extent, explain the origin of attractive energies between spike proteins, in their *up* prefusion state, and host-cell receptors. We implemented a fragment-based quantum-biochemical method that evaluates the strength and detailed nature, i.e. attractive or repulsive, of ACE2 interactions with S-protein *receptor binding domains*. We used a widely cited SARS-CoV-1...hACE2 crystallographic structure^3^ to perform such fragment-based calculations that clearly identify which contact residue fragments give rise to the attractive hACE2...S-RBD interaction energy and, therefore, promote viral infection.

The *receptor binding motif* (S-RBM) of spike proteins, an integral and main functional component of their S-RBD, is at the interface which potentially binds to a host receptor such as hACE2. Importantly, despite a sequence identity of about 72-73$\%$ between the *domains* (S-RBD) of SARS-CoV-1 and SARS-CoV-2, the identity of their respective *motifs* (S-RBM) is significantly lower, only about 47.8$\%$^28^. Thus, although structural similarities may produce *some* similar interaction mechanisms between the S-RBD of SARS-CoV-1 and SARS-CoV-2 with hACE2, their S-RBM structural differences^7^ will likely produce other, concomitant but different, attractive or repulsive hACE2...S-RBD interactions. To develop therapeutic drugs and to understand the action of antibodies^29^ which target viral spike proteins, it is useful to study each specific viral S-RBD and their interactions with hACE2. In this work we focus on identifying the main, molecular level, interactions between the S-RBD of SARS-CoV-1, a potentially reemerging public health threat, and hACE2.

The ability of coronaviruses to recognize their host-cell receptors is a first and crucial determinant of their host range and infectivity. It has been realized that the process of recognition is not due to accidental or random intermolecular events but to viral-RBD and host-receptor structural complementarity^30^. Less attention has been paid, however, to specific and concomitant energetic complementarities which favor non-covalent attraction at the viral-host interface. Here, we establish a quantitative link between structural complementarity and concomitant physico-chemical viral-host non-covalent interactions. We implemented a fragment-based quantum biochemical method to study the hACE2...S-RBD interface. We report, in units of kcal/mol, the *total* interaction energy between contact residues of hACE2 and the SARS-CoV-1 S-RBD. In addition we evaluate *partial* interaction energies between specific sets of four hACE2 residues, herein called *quartets*, with their neighboring S-RBD residues. Thus, we identify which hACE2 *quartets* are attractive and which are repulsive relative to the SARS-CoV-1 S-RBD. Likewise, we identify which S-RBD residue *quartets* are attractive or repulsive relative to the hACE2 receptor. Our results enhance the understanding of molecular level mechanisms of hACE2 and S-RBD recognition and, in addition, identify potential therapeutic targets and SARS-CoV-1 epitopes.

**Table 1:** Energies$^{\mathrm{a}}$ of the human receptor (hACE2), spike protein binding domain (S-RBD) and their interaction energies without ($\hbox {E}_{\mathrm{Int}}^{\mathrm{DFT}}$) and with ($\hbox {E}_{\mathrm{Int}}^{\mathrm{DFT-DD}}$) van der Waals *dispersion* corrections [DD]$^{\mathrm{b}}$.

$\hbox {E}_{\mathrm{hACE2...S-RBD}}$	$\text {E}_{\text {hACE2}}$	$\text {E}_{\text {S-RBD}}$	$\text {E}_{\text {Int}}^{\text {DFT}}$	$\text {E}_{\text {Int}}^{\text {DFT}}$	$\text {E}_{\text {Int}}^{\text {DD}}$	$\text {E}_{\text {Int}}^{\text {DFT-DD}}$
[Hartrees]	[Hartrees]	[Hartrees]	[Hartrees]	[kcal/mol]	[kcal/mol]	[kcal/mol]
$-$22,240.4565923	$-$10,953.2725200	$-$11,287.1426313	$-$0.0414410	$-$26.00	$-$378.26	$-$404.26

$^{\mathrm{a}}$DFT energies computed with the B3LYP^31^ functional and 6-31+G* basis in the gas-phase.

$^{\mathrm{b}}$Distance-dependent (DD) *dispersion* evaluated with the B3LYP-DD semiempirical method^27^.

## Results

Total and partial interaction energies between hACE2 and the SARS-CoV-1 S-RBD were computed, in the low temperature limit, *via* quantum biochemical calculations and the supermolecular approach^27^. A fragment-based methodology, by which proteins are divided into fragments, was used to evaluate *partial* interaction energies and identify the dominant, attractive or repulsive, sets of residues at the hACE2-S-RBD interface. All calculations were based on all-electron dispersion-corrected^27^ density functional theory^31,32^.

### Attractive nature of the hACE2...S-RBD interaction

Table 1 shows that the net interaction between hACE2 and the S-RBD is attractive as indicated by the negative sign of their interaction energy ($\text {E}_{\text {Int}}^{\text {DFT-DD}}$) . This finding confirms and is consistent with the tendency of the SARS-CoV-1 prefusion S-RBD to bind to the hACE2 receptor^10,14^. The attractive nature of the interaction energy is also consistent with the structure of the virus-receptor interface, as displayed by the crystallographic structure^3^, which corresponds to a thermodynamically favored conformation.

The hACE2...S-RBD interaction energy was calculated, separately, in gas and solvent phases with both results corresponding to a net intermolecular attraction. In addition van der Waals *dispersion* corrections were evaluated *via* the accurate B3LYP-DD methodology^27^ which, in the gas phase, added a significant attractive contribution. The gas-phase interaction energies, in the absence and presence of *dispersion* corrections, were on the order of $-$26 kcal/mol and $-$404 kcal/mol, respectively, when evaluated with the 6-31+G* basis set (Table 1). Similar trends were found from calculations with other basis sets as shown in Supplementary Table S1. It should be noted that *partial* electrostatic contributions to the interaction energy can be attractive or repulsive which tends to lower the net additive magnitude of this mechanism. By contrast, *dispersion* contributions are additively attractive which explains the large energetic contribution of *dispersion* ($\text {E}_{\text {Int}}^{\text {DD}}$). *Dispersion* contributions were calculated at the short intermolecular distances corresponding to hACE2...S-RBD noncovalent attachment as displayed by the crystallographic structure^3^. At these short distances van der Waals forces are particularly strong.

In contrast to gas-phase *dispersion*-corrected interaction energies ($\text {E}_{\text {Int}}^{\text {DFT-DD}}$), which in that limit are generally accurate to better than 1 kcal/mol^27^, the calculation of solvent-phase interaction energies introduces greater uncertainties. Thus, the solvent-phase energies given in Supplementary Table S1 should be considered as rough approximations which illustrate the still attractive, although weaker, hACE2...S-RBD intermolecular interactions when solvation effects are taken into account.

**Figure 2:**
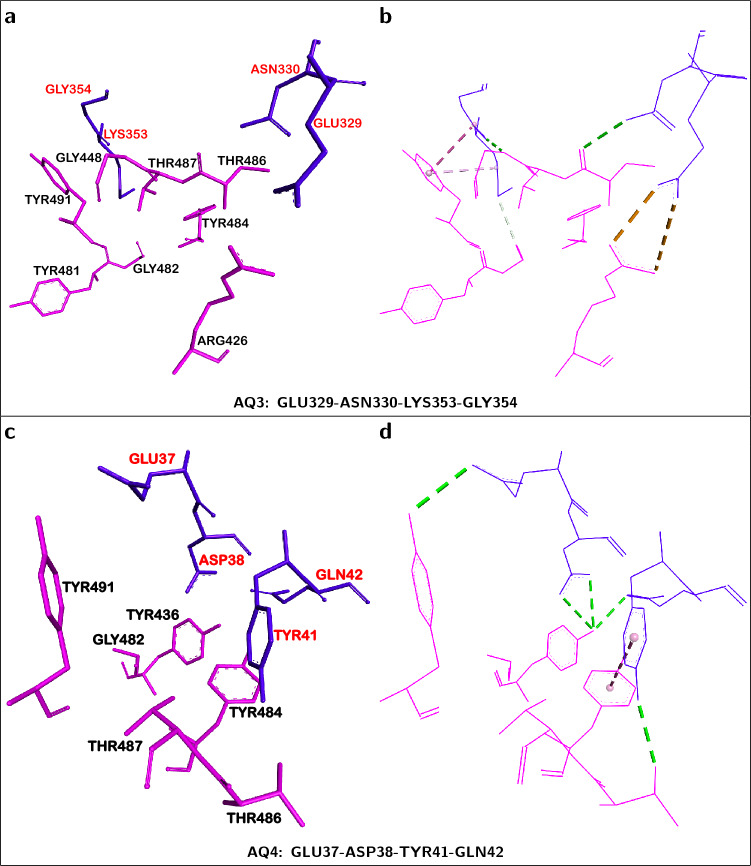
The two hACE2-centered fragments producing a net *attractive* interaction towards S-RBD. ACE2 *quartet* residues (shown in blue) and neighboring S-RBD residues (shown in pink) corresponding to the dominant *attractive* ACE2...S-RBD interactions. ACE2 is the structural reference. (**a, b**) Constituent residues and selected intermolecular interactions, respectively, of *attractive* **AQ3**-centered fragment. (**c, d**) Constituent residues and selected intermolecular interactions, respectively, of *attractive* **AQ4**-centered fragment. Dotted lines correspond to i) amid-$\pi$ interactions in **(b)** and $\pi$-$\pi$ interactions in **(d)** (dotted pink lines), ii) conventional (dotted green lines) and non-conventional (dotted white lines) hydrogen bonds and iii) electrostatic interactions (dotted yellow lines).

**Table 2:** Human ACE2 receptor (hACE2) *quartets* and their interaction energies [kcal/mol]$^{\mathrm{a}}$with neighboring$^{\mathrm{b}}$ S-RBD residues.

Quartet	Human ACE2 Receptor			
	Residues	$\text {E}_{\text {Int}}^{\text {DFT}}$	$\text {E}_{\text {Int}}^{\text {DD}}$	$\text {E}_{\text {Int}}^{\text {Total}}$
AQ1	ASP30-LYS31-ASN33-HIS34	$+$17.50	$-$13.10	$+$4.41
AQ2	GLN24-ALA25-LYS26-THR27	$+$51.12	$-$7.32	$+$43.80
AQ3	GLU329-ASN330-LYS353-GLY354	$-$30.82	$-$28.81	$-$59.63
AQ4	GLU37-ASP38-TYR41-GLN42	$-$38.48	$-$16.30	$-$54.78
AQ5	LEU91-THR92-GLN325-GLY326	$+$11.82	$-$2.25	$+$9.57
AQ6	MET82-TYR83-GLN89-ASN90	$+$34.46	$-$5.23	$+$29.23
AQ7	SER44-LEU45-ALA46-SER47	$+$28.01	$-$2.60	$+$25.41
AQ8	SER77-THR78-LEU79-ALA80	$+$26.03	$-$2.01	$+$24.02

^a^DFT energies computed at 6-311+G(d,p)/B3LYP level; *Dispersion* (DD) corrections evaluated with semiempirical method^27^.

^b^All S-RBD residues within 4.5 $\AA$ of each ACE2 *quartet* were included.

**Figure 3:**
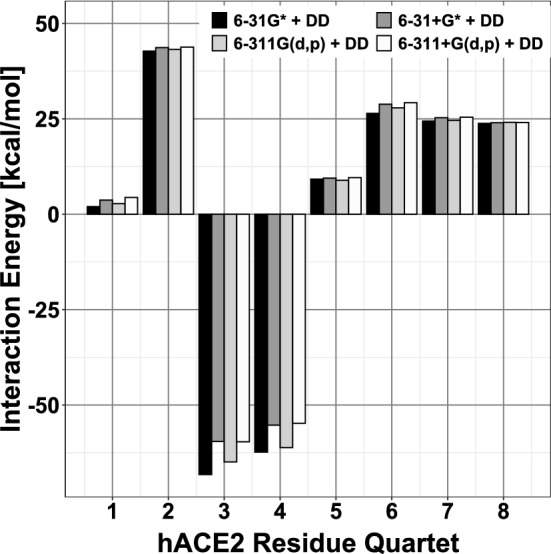
Partial energies of hACE2 interaction with the S-RBD. Main repulsive (positive) and attractive (negative) interaction energies [kcal/mol] between hACE2 *quartets*, used as structural references, and neighboring SARS-CoV-1 S-RBD residues. The four adjacent vertical bars for each *quartet* correspond, from left to right, to *dispersion*-corrected [DD]^27^ evaluations with the 6-31G*, 6-31+G*, 6-311G(d,p) and 6-311+G(d,p) basis sets.

**Figure 4:**
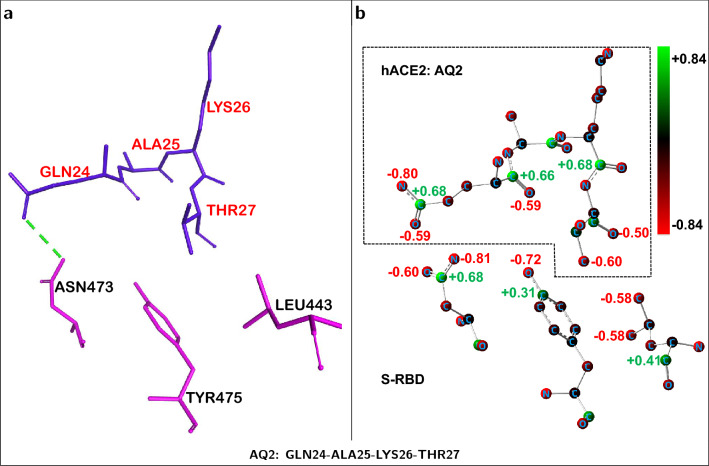
hACE2-centered fragment producing a net *repulsive* interaction towards S-RBD. (**a**) ACE2 *quartet* residues (shown in blue) and neighboring S-RBD residues (shown in pink) corresponding to a *repulsive* ACE2...S-RBD interaction. ACE2 is used as the structural reference. (**b**) Corresponding residue atoms displaying positive charge (green spheres) or negative charge (red spheres). Atomic partial charges, evaluated with NBO^33^ method, for selected atoms are shown. The electrostatic hACE2...S-RBD interface, for this fragment, is dominated by negatively charged (red) atoms leading to a net intermolecular repulsion. Positively charged hydrogen atoms not shown.

### Structural separation of the hACE2...S-RBD interface into quartet residue fragments

**Figure 5:**
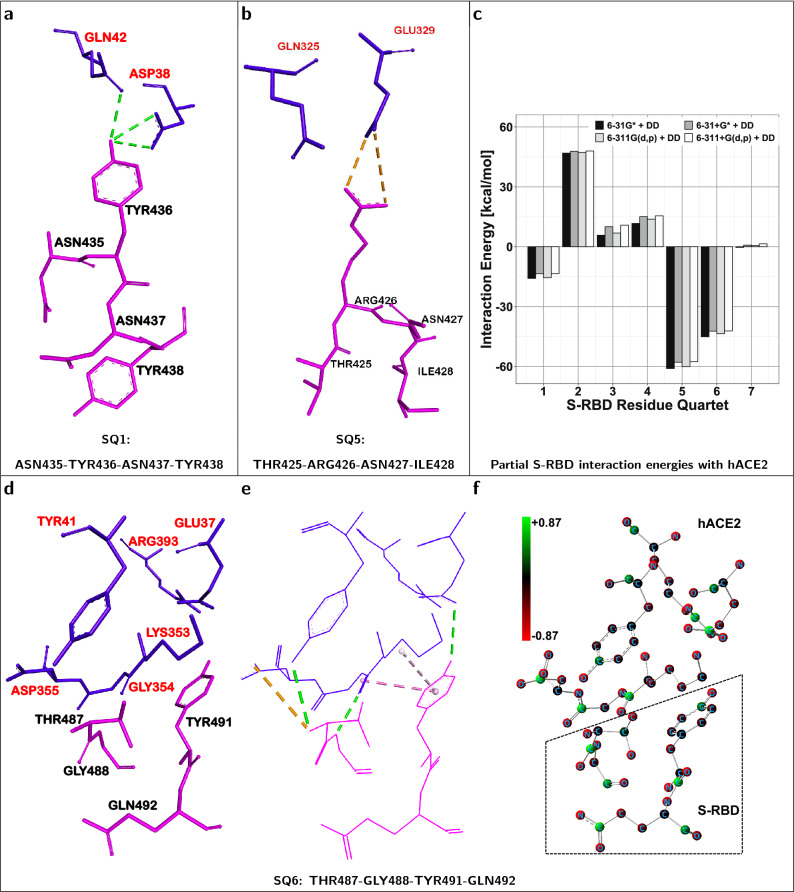
The three S-RBD-centered fragments producing a net *attractive* interaction towards hACE2. (**a, b**) S-RBD *quartet* residues (shown in pink) and neighboring ACE2 residues (shown in blue) corresponding to *attractive* ACE2...S-RBD interactions. S-RBD is the structural reference. (**c**) Main repulsive (positive) and attractive (negative) interactions [kcal/mol] between *quartets* of the SARS-CoV-1 S-RBD, used as structural references, and neighboring residues of the human hACE2 receptor. The four adjacent vertical bars for each *quartet* correspond, from left to right, to *dispersion*-corrected [DD]^27^ interaction energies evaluated with the 6-31G*, 6-31+G*, 6-311G(d,p) and 6-311+G(d,p) basis sets. (**d, e**) Constituent residues and selected intermolecular interactions, respectively, of *attractive* **SQ6**-centered fragment. Dotted lines correspond to i) amid-$\pi$ interactions (dotted pink lines), ii) conventional hydrogen bonds (dotted green lines) and iii) electrostatic interactions (dotted yellow lines). (**f**) Corresponding atoms with positive (green spheres) or negative (red spheres) partial charge. Positively charged hydrogen atoms not shown. Charges evaluated with NBO^33^ method.

It is of great interest to identify the dominant sets of contact residues involved in physico-chemical attraction or repulsion between hACE2 and the S-RBD. It was determined that partitioning each protein structure into sets of four residues allowed for a qualitatively meaningful determination of intermolecular interaction energies. Protein fragments of smaller size did not include a minimum of nearest-neighbor and next-nearest-neighbor interactions between protein residues to allow for meaningful estimates of *partial* contributions to the overall hACE2...S-RBD interaction energy. Therefore, sets of four ACE2 contact residues, herein referred to as ACE2 *quartets*, were selected together with their neighboring, i.e. within a range of 4.5 $\AA$, viral S-RBD residues. An hACE2-centered *supermolecular fragment* is herein defined as a particular hACE2 residue *quartet* and its neighboring S-RBD residues. Thus, any S-RBD residue localized in a region of strong noncovalent interaction with a particular ACE2 *quartet* was included in a respective *supermolecular fragment* of the hACE2...S-RBD complex. Examples of such hACE2...S-RBD *supermolecular fragments* are shown in Fig. 2. These structural constructs were then used to compute *partial*, attractive or repulsive, interaction energies between particular hACE2 *quartets* and the S-RBD as reported in Table 2 and Fig. 3. Similarly, an S-RBD-centered *supermolecular fragment* constitutes a particular S-RBD residue *quartet* and its neighboring hACE2 residues with examples given in Fig. 5.

### Evaluation of partial, attractive or repulsive, hACE2...S-RBD interactions

The net attractive intermolecular interaction promotes the thermodynamic stability of the hACE2...S-RBD complex. Despite the net interaction being attractive, the calculated interaction energies ($\text {E}_{\text {Int}}^{\text {DFT-DD}}$) can be interpreted as the combined result of several *partial* interactions, some attractive and some repulsive, between particular sets of hACE2 and S-RBD residues. The evaluation of *quartet*-centered *partial* contributions to the interaction energy allow the identification, as illustrated by Fig. 1, of which protein fragments are primarily responsible for binding of the ACE2...S-RBD complex. In addition evaluation of *partial* interactions between hACE2 and S-RBD fragments, whether of attractive or repulsive character, provide molecular-level and energetic insight about the related processes of host-virus recognition and attachment.

Our results show that some *supermolecular fragments* at the ACE2...S-RBD interface are intrinsically attractive and thus directly favor the human receptor’s complexation with the virus S-protein. Although other supermolecular fragments were found to be intrinsically repulsive, these too play a concomitant and important role in the formation of the ACE2...S-RBD complex. In fact the repulsive fragments, together with their attractive counterparts, help to guide the process of intermolecular recognition which ultimately leads to attachment. Among the supermolecular fragments that produce attractive hACE2...S-RBD interactions, *dispersion* forces were also found to play an important role. The latter correspond to *partial* contributions to the *dispersion* energy and are consistent with the importance of the van der Waals mechanism previously uncovered for the total interaction energy (Table 1) of the entire host-virus contact interface.

### Identification of key hACE2-centered quartet interactions with S-RBD

Table 2 and Fig. 3 display *partial* interaction energies between hACE2 *quartets* and their neighboring S-RBD residues. There are two ACE2 *quartets*, **AQ3** (GLU329-ASN330-LYS353-GLY354) and **AQ4** (GLU37-ASP38-TYR41-GLN42), whose interactions with S-RBD are strongly attractive as indicated by the magnitudes and negative signs, $-$59.63 and $-$54.78 kcal/mol, respectively, of their interaction energies. Fig. 2 shows the structural composition of these two ACE2 *quartets* and their closely interacting S-RBD residues. The physico-chemical origin of the attractive nature of their *partial* ACE2...S-RBD interactions is not only related to conventional electrostatic effects, including hydrogen bonding, but also to sizable *dispersion* contributions (Table 2). For *quartets* **AQ3** and **AQ4** *dispersion* contributions were on the order of $-$28.81 and $-$16.30 kcal/mol, respectively, corresponding to $\approx$48$\%$ and $\approx$30$\%$ of their *partial* interaction energies. Additional electronic structure calculations were done using the same protocol but using other, closely related, computational basis sets. Supplementary Tables S2-S4 list the corresponding energies which display similar trends, thus confirming the intrinsically attractive nature of ACE2 *quartets* **AQ3** and **AQ4** with respect to S-RBD.

The quantum mechanical (*ab-initio*) character of the present calculations takes into account, at the same time, intermolecular interactions in the low temperature regime. Therefore, the present calculations do not separate or distinguish, contrary to traditional classifications, between particular types of intermolecular forces with the exception of *dispersion* contributions to van der Waals forces. However, qualitatively, it is possible to relate some of our results to traditional classifications. To this effect, Fig. 2 shows some qualitative (color coded) assignments which include: i) amid-$\pi$ interactions in **(b)** and $\pi$-$\pi$ interactions in **(d)** (dotted pink lines) which, involving six-membered aromatic rings, more fundamentally correspond in the present work to *dispersion* forces; ii) conventional (dotted green lines) and non-conventional (dotted white lines) hydrogen bonds; and iii) electrostatic interactions (dotted yellow lines).

Despite the overall ACE2...S-RBD interaction as well as the dominant *partial* contributions being attractive, Fig. 3 also shows that several of the ACE2 *quartets* are actually repulsive relative to the S-RBD. ACE2 *quartet* **AQ2** is the most repulsive with a *partial* interaction energy of about $+$43.80 kcal/mol (Table 2) which includes a large repulsive contribution ($\approx +$51.12 kcal/mol) and only a small ($\approx -$7.32 kcal/mol) *dispersion* component. The molecular structure and atomic partial charge distribution of the corresponding *supermolecular fragment* are shown in Fig. 4. The intermolecular interface of this fragment is rich in negatively charged atoms (not including hydrogen atoms) with both types of interface residues, belonging to hACE2 and the S-RBD, displaying several negative partial charges. This indicates that the repulsive interaction energy, intrinsic to this fragment, is primarily due to electrostatic repulsion.

### Identification of key S-RBD-centered quartet interactions with hACE2

Table 3 and Figure 5c show *partial* energies corresponding to spike protein (S-RBD) *quartets* interacting with neighboring hACE2 residues. There are two S-RBD *quartets*, **SQ5** (THR425-ARG426-ASN427-ILE428) and **SQ6** (THR487-GLY488-TYR491-GLN492), which dominate the attractive interactions with hACE2 and lead to *partial* interaction energies of $-$57.57 and $-$42.15 kcal/mol, respectively. Consistent with the absence of six-membered rings no significant *dispersion* contribution was evaluated for *quartet* **SQ5**. However, for the opposite reason, *dispersion* contributions were more prominent in the interaction energy of *quartet* **SQ6** ($\approx -28\hbox { kcal/mol}$) which, as illustrated in Fig. 5e, displays interactions associated with a six-membered TYR491 ring. Consistent with its relatively weak non-*dispersion* contribution ($\approx$-14 kcal/mol), Fig. 5f shows that the **SQ6**-centered fragment does not have a strongly dominant set of atomic partial charges, of either positive or negative sign, at its interface. Since both types of atoms, positively charged and negatively charged, are present at the interface of this fragment, leading to a complex combination of attractions and repulsions, the net electrostatic effect is only moderately attractive. In addition, S-RBD *quartet* **SQ1** (ASN435-TYR436-ASN437-TYR438) produced a substantially weaker attraction relative to hACE2. Additional electronic structure calculations were done with the same protocol but using other, closely related, computational basis sets. Supplementary Tables S5-S7 list the corresponding energies which, displaying similar trends, confirm a dominant and intrinsically attractive nature of S-RBD *quartets* **SQ5** and **SQ6** with respect to hACE2. The weaker attractive nature of S-RBD *quartet* **SQ1** was also confirmed by the data in the Supplementary Tables.

**Figure 6:**
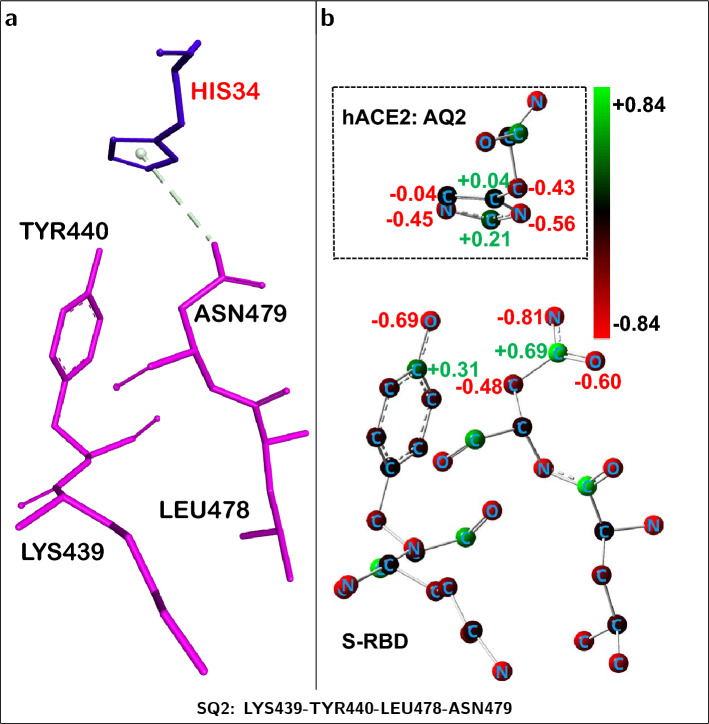
S-RBD-centered fragment producing a net *repulsive* interaction towards hACE2. (**a**) S-RBD *quartet* residues (shown in pink) and neighboring ACE2 residues (shown in blue) corresponding to a dominant *repulsive* ACE2...S-RBD interaction. S-RBD is used as the structural reference. (**b**) Corresponding residue atoms displaying positive partial charge (green spheres) or negative partial charge (red spheres). Atomic partial charges, evaluated with NBO^33^ method, for selected atoms are shown. The electrostatic hACE2...S-RBD interface, for this fragment, is dominated by negatively charged (red) atoms leading to a net intermolecular repulsion. Positively charged hydrogen atoms not shown.

Table 3 and Fig. 5c identify S-RBD *quartet* **SQ2** as the most repulsive relative to hACE2 ($+$47.95 kcal/mol). The repulsive component of its interaction energy ($\approx$ $+$52 kcal/mol) dominates the character of the corresponding fragment which only displays a minor attractive contribution. Fig. 6b shows that the electrostatic interface of the fragment is rich in negatively charged atoms which largely explains its net repulsive character.

**Table 3:** S-RBD-centered *quartets* and their interaction energies [kcal/mol]$^{\mathrm{a}}$ with neighboring$^{\mathrm{b}}$ hACE2 residues.

Quartet	SARS-CoV-1 S-RBD			
	Residues	$\text {E}_{\text {Int}}^{\text {DFT}}$	$\text {E}_{\text {Int}}^{\text {DD}}$	$\text {E}_{\text {Int}}^{\text {Total}}$
SQ1	ASN435-TYR436-ASN437-TYR438	$-$10.05	$-$3.40	$-$13.45
SQ2	LYS439-TYR440-LEU478-ASN479	$+$52.04	$-$4.09	$+$47.95
SQ3	PHE483-TYR484-THR485-THR486	$+$31.72	$-$20.90	$+$10.81
SQ4	PRO470-ALA471-LEU472-ASN473	$+$24.48	$-$8.99	$+$15.49
SQ5	THR425-ARG426-ASN427-ILE428	$-$55.25	$-$2.32	$-$57.57
SQ6	THR487-GLY488-TYR491-GLN492	$-$14.06	$-$28.08	$-$42.15
SQ7	TYR442-LEU443-TYR475-TRP476	$+$18.51	$-$17.02	$+$1.48

^a^DFT energies computed at 6-311+G(d,p)/B3LYP level; *Dispersion* (DD) corrections evaluated with semiempirical method^27^.

^b^All ACE2 residues within 4.5 $\AA$ of each S-RBD *quartet* were included.

## Discussion

### Relationship between biomolecular structure and quantum-mechanical non-covalent hACE2...S-RBD interactions

Interaction energies, as defined in Eqs. (2-3), can be positive or negative and are a measure of the tendency of two biomolecular structures to repel or attract each other, respectively. This idea is supported by the relationship between interaction energies, for non-covalent intermolecular interactions, and corresponding changes in enthalpy. In addition our density-functional frozen-geometry estimates, for the individual attractive fragments, suggest that although entropy contributions produce corresponding Gibbs free energy changes of somewhat higher (more positive) numerical value, their trends are similar to those reported here for the corresponding interaction energies. Within the present quantum-biochemical framework interaction energies are the combined result of several physico-chemical effects, incorporated in Eqs. (1-3), some of which are intrinsically attractive whereas others are repulsive. For example, intermolecular Coulomb interactions between atoms whose charge has the same(different) sign are repulsive(attractive), respectively, whereas intermolecular *dispersion* van der Waals forces are additively attractive. *Dispersion* forces correspond to the attractive portion of intermolecular van der Waals potentials^27^ and were carefully evaluated and incorporated in this work.

The structural details, at the molecular level, of host-virus interfaces are crucial for determining the strength and relative importance of the various types of intermolecular forces since these are dependent on different powers of interatomic distances ($r_{ij}$). For example, Coulomb interaction energies between two atomic centers i and j, separated by a distance $r_{ij}$, scale as $1\over r_{ij}$. By contrast, at short intermolecular distances (i.e. the nonretarded regime), attractive *dispersion* contributions to van der Waals energies scale inversely to the sixth power ($1\over r_{ij}^6$) of the distances^27,34,35^. Thus, the relative importance of each type of noncovalent intermolecular interaction is highly dependent on intermolecular distances with Coulomb interactions being longer range and *dispersion* interactions playing critical roles at shorter ranges. In this work we focus on evaluation of host-virus interactions corresponding to the intermolecular distances of the non-covalently bound hACE2...S-RBD structure determined by crystallography^3^. That is, we focus on key hACE2...S-RBD interactions at the crucial structural, as opposed to temporal, stage when hACE2 has formed, upon completion of a process of intermolecular recognition, a thermodynamically favored non-covalent complex with the prefusion conformation of the SARS-CoV-1 spike protein.

### Particularly important attractive residues at the hACE2...S-RBD interface

The evaluation of two sets of interaction energies, hACE2-centered *quartets* interacting with S-RBD and S-RBD-centered *quartets* interacting with hACE2, allows the identification of contact residues of particular importance to the host-virus binding energy. Tables 2 and 3 provide complementary information and suggest a number of residues which dominate the hACE...S-RBD attractive energy. Most hACE2 residues belonging to *quartet* **AQ3** (GLU329, LYS353 and GLY354) and all hACE2 residues making up *quartet* **AQ4** (GLU37, ASP38, TYR41 and GLN42) are involved in significant attractive interactions as determined by both, hACE2-centered and S-RBD-centered, energetic calculations. Similarly, residue TYR436 from S-RBD *quartet* **SQ1**, residue ARG426 from S-RBD *quartet* **SQ5** and most residues from S-RBD *quartet* **SQ6** (THR487, GLY488 and TYR491) are likely primary attractors, with respect to hACE2, based on a similar analysis.

Some of the previous results are consistent not only with available crystallographic data but also with functional and substitutional studies. For example the strong (salt bridge) interaction between hACE2(GLU329) and S-RBD(ARG426) has been noticed^22^ from structural analysis whereas the importance, for hACE2 binding, of S-RBD residues ARG426 and THR487 was suggested from mutation substitutional studies^28^. In addition, S-RBD residue TYR484 has been postulated as an important hACE2 binder^3,28^. In this work this residue is part of S-RBD *quartet* **SQ3** which produces a net weak repulsion relative to hACE2. However Table 3 shows that, due to the presence of its phenolic group, TYR484 likely contributes an attractive *dispersion* interaction consistent with the $\approx$ −29.90 kcal/mol *dispersion* energy of the entire *quartet*. Thus, this residue can potentially be an important attractor even though the evidence in the present study is somewhat indirect.

## Conclusion

SARS-CoV-1 is a potentially-reemerging^1,2^ highly-pathogenic virus and substantial gaps remain in our understanding of its molecular-level mechanisms of transmissibility^2^. Spike proteins of coronaviruses interact, *via* their *receptor binding domains*, with human ACE2 receptors. The identification of protein fragments, at the hACE2...S-RBD interface, which are primarily responsible for close-range attractive or repulsive interactions is of importance i) fundamentally for elucidating the physico-chemical origin of host-virus attachment and ii) for identifying specific therapeutic targets and viral epitopes. Among the various anti-coronavirus therapeutic strategies there are two which may, in particular, benefit from this study. Namely, therapies which target the human ACE2 receptor and therapies which attempt to block SARS-CoV-1 spike proteins. The present studies, complementary to those based on X-ray crystallography or cryo-EM, have identified which protein fragments, herein referred to as residue *quartets*, are involved in the strongest, attractive or repulsive, hACE2...S-RBD interactions. The dominant residue *quartets* of attractive nature are shown in Fig. 1.

Our results are based on three-dimensional structures of the human ACE2 receptor and SARS-CoV-1 spike protein. The present identification of specific, attractive and repulsive, biomolecular fragments as well as the quantification of their interaction energies is particular to this system, namely hACE2 interacting with the prefusion conformation of the SARS-CoV-1 spike protein. Our results suggest interaction mechanisms of hACE2 with other similar, but not structurally identical, spike protein RBDs such as those from SARS-CoV-2. The fact that the sequence identity of the *domains* (S-RBD) from SARS-CoV-1 and SARS-CoV-2 is about 72-73$\%$ whereas the identity of their *motifs* (S-RBM) is only about 48$\%$^28,36^ suggests similarities as well as differences in the relative importance of their specific amino acid residues towards hACE2 binding energies. This would be consistent with structural differences between their respective S-RBM and their non-identical binding affinities towards hACE2^7^. Studies of hACE2 with SARS-CoV-2 must take into account the sequence and structural details of its own S-RBD. Indeed, some key hACE2-interacting S-RBD residues in SARS-CoV-1 may not play an equivalent role in SARS-CoV-2^37^. Interaction energy studies for SARS-CoV-2 to determine similarities and differences in hACE2...S-RBD binding, relative to SARS-CoV-1, are currently in progress in our laboratory.

## Methods

The biomolecular structure of the hACE2...S-RBD interface corresponding to the SARS-CoV-1 virus, as extracted from the published X-ray crystallographic structure^3^, was studied as a single structure and also separated into *quartet*-based fragments as described in the main text. A locally developed algorithm was used to divide the interacting hACE2...S-RBD molecular structure into *quartet* fragments. It was determined that, either hACE2-centered or S-RBD-centered fragments composed of at least four residues was necessary to evaluate fragment-based interaction energies. Fragments of smaller size, i.e. containing *quartets* of less than four residues, did not include a minimum of nearest neighbor and next nearest neighbor interactions to provide reliable qualitative estimates of *partial* intermolecular interaction energies.

All electron Khon-Sham density functional calculations were done on the overall structure in both, gas and solvent, phases. Similar calculations were done on all host-virus biomolecular fragments which in the main text are referred to as *supermolecular fragments*. Khon-Sham density functional calculations solve, numerically, a quantum mechanical Hamiltonian that includes an approximation to the exact, but unknown, exchange-correlation potential. Energies were obtained, in the low temperature limit, in terms of Eq. (1) for all biomolecular structures described in the text.

1 $$\begin{aligned} \hbox {E}^{\mathrm{DFT}}&= {KE}[\rho (\mathbf{r})] + {1\over 2}\int {\rho (\mathbf{r}) \rho (\mathbf{r}^\prime ) \over | \mathbf{r} - \mathbf{r}^\prime |} \hbox {d}{} \mathbf{r} \hbox {d}{} \mathbf{r}^\prime \nonumber \\&+ \hbox {E}_{xc}[\rho (\mathbf{r})] + \int \hbox {v}(\mathbf{r}) \rho (\mathbf{r}) \hbox {d}{} \mathbf{r} + \text {E}_{\text {NN}} \end{aligned}$$

Here, $\rho (\mathbf{r})$ represents the electron density obtained from solution of the Khon-Sham equations. The B3LYP^31,32^ exchange-correlation functional was used in the energy calculations due to its complementarity with the B3LYP-DD *dispersion*-correction methodology^27^. Many exchange-correlation functionals, including B3LYP, fail to properly account for intermolecular *dispersion* van der Waals contributions. Therefore, semiempirical corrections ($\text {E}_{\text {Int}}^{\text {DD}}$) were added to the Khon-Sham interaction calculations *via* the B3LYP-DD methodology^27^ which fairly accurately incorporates *dispersion* for a range of intermolecular distances. As reported in the main text and the Supplementary Tables, several basis sets of progressively increasing size [including 6-31G*, 6-31+G*, 6-311G(d,p) and 6-311+G(d,p)] were used in a series of independent energy calculations to ensure qualitative consistency of the numerical results. Interaction energies were computed in the absence ($\text {E}_{\text {Int}}^{\text {DFT}}$) and presence ($\text {E}_{\text {Int}}^{\text {DFT-DD}}$) of *dispersion*, *via* Eqs. (2-3), following the supermolecular approach as described in the B3LYP-DD reference^27^. Atomic partial charges were computed with the Natural Bond Orbital (NBO) method^33^ as implemented in the Gaussian package^38^ which, for the basis sets used in this work, generally show consistent results.

2 $$\begin{aligned} \hbox {E}_{\mathrm{Int}}^{\mathrm{DFT}}&= \hbox {E}_{\mathrm{hACE2...S-RBD}}^{\mathrm{DFT}}-\hbox {E}_{\mathrm{hACE2}}^{\mathrm{DFT}}-\hbox {E}_{\mathrm{S-RBD}}^{\mathrm{DFT}} \end{aligned}$$

3 $$\begin{aligned} \hbox {E}_{\mathrm{Int}}^{\mathrm{DFT-DD}}&= {} \hbox {E}_{\mathrm{Int}}^{\mathrm{DFT}} + \hbox {E}_{\mathrm{Int}}^{\mathrm{DD}} \end{aligned}$$

## Supplementary information

Supplementary Tables.
