# Supplementary material for: Contact residue contributions to interaction energies between SARS-CoV-1 spike proteins and human ACE2 receptors
Source: Sci Rep. 2021 Jan 13;11:1156. doi: 10.1038/s41598-020-80942-6 (PMC7806713; doi:10.1038/s41598-020-80942-6)
Supplement: Supplementary file 1 — Supplementary Tables. [file 41598_2020_80942_MOESM1_ESM.pdf]

Supplementary Material

Contact residue contributions to interaction energies between SARS-CoV-1 spike proteins and human ACE2 receptors

Jorge H. Rodriguez* and Akshita Gupta

Computational Biomolecular Physics Group, Department of Physics and Astronomy

Purdue University, West Lafayette, IN 47907-2036, USA

TABLE S1. | **Energies[a] of the human receptor (hACE2), spike protein binding domain (S-RBD) and their interaction energies without (E_{Int}^{DFT}) and with (E_{Int}^{DFT-DD}) van der Waals *dispersion* corrections [DD].[b]**

Method	$E_{hACE2...S-RBD}$ [Hartrees]	E_{hACE2} [Hartrees]	E_{S-RBD} [Hartrees]	E_{Int}^{DFT} [Hartrees]	E_{Int}^{DFT} [kcal/mol]	E_{Int}^{DD} [kcal/mol]	E_{Int}^{DFT-DD} [kcal/mol]
			Gas Phase				
6-31G*	-22239.5174586	-10952.8147793	-11286.6497266	-0.0529527	-33.23	-309.29	-342.52
6-31+G*	-22240.4565923	-10953.2725200	-11287.1426313	-0.0414410	-26.00	-378.26	-404.26
6-311G(d,p)	-22244.913869	-10955.4641059	-11289.4000815	-0.0496816	-31.18	-309.29	-340.46
			Solvent Phase				
6-31G* [CPCM][c]	-22240.2390122	-10953.2392017	-11286.9968304	-0.0029801	≈-1.87		
6-31G* [SMD][d]	-22240.6739549	-10953.4525363	-11287.2151241	-0.0062945	≈-3.95		

[a] DFT energies computed with the B3LYP [1] functional.
[b] Distance-dependent *dispersion* (DD) corrections evaluated with the B3LYP-DD semiempirical method. [2]
[c] Solvation effects included *via* the CPCM method. [3]
[d] Solvation effects included *via* the SMD method. [4]

TABLE S2. | **Human ACE2 receptor (hACE2) *quartets* and their interaction energies [kcal/mol][a] with neighboring[b] virus S-RBD residues.**

Quartet	**Human ACE2 Receptor Residues**	E_{Int}^{DFT}	E_{Int}^{DD}	E_{Int}^{Total}
AQ1	ASP30-LYS31-ASN33-HIS34	+14.78	−12.78	+2.00
AQ2	GLN24-ALA25-LYS26-THR27	+49.68	−6.95	+42.73
AQ3	GLU329-ASN330-LYS353-GLY354	−42.27	−25.97	−68.24
AQ4	GLU37-ASP38-TYR41-GLN42	−47.29	−15.07	−62.37
AQ5	LEU91-THR92-GLN325-GLY326	+11.41	−2.24	+9.18
AQ6	MET82-TYR83-GLN89-ASN90	+31.07	−4.65	+26.42
AQ7	SER44-LEU45-ALA46-SER47	+26.97	−2.57	+24.40
AQ8	SER77-THR78-LEU79-ALA80	+25.81	−1.99	+23.82

[a] DFT energies computed at 6-31G*/B3LYP level; Dispersion (DD) corrections evaluated with semiempirical method. [2]
[b] All S-RBD residues within 4.5 Å of ACE2 *quartet* were considered.

TABLE S3. | **Human ACE2 receptor (hACE2) *quartets* and their interaction energies [kcal/mol][a] with neighboring[b] virus S-RBD residues.**

Quartet	**Human ACE2 Receptor Residues**	E_{Int}^{DFT}	E_{Int}^{DD}	E_{Int}^{Total}
AQ1	ASP30-LYS31-ASN33-HIS34	+16.80	−13.10	+3.70
AQ2	GLN24-ALA25-LYS26-THR27	+51.00	−7.32	+43.68
AQ3	GLU329-ASN330-LYS353-GLY354	−30.76	−28.81	−59.57
AQ4	GLU37-ASP38-TYR41-GLN42	−38.97	−16.30	−55.28
AQ5	LEU91-THR92-GLN325-GLY326	+11.71	−2.25	+9.46
AQ6	MET82-TYR83-GLN89-ASN90	+34.05	−5.23	+28.82
AQ7	SER44-LEU45-ALA46-SER47	+27.89	−2.60	+25.29
AQ8	SER77-THR78-LEU79-ALA80	+26.00	−2.01	+23.99

[a] DFT energies computed at 6-31+G*/B3LYP level; Dispersion (DD) corrections evaluated with semiempirical method. [2]
[b] All *concap* S-RBD residues within 4.5 Å of ACE2 *quartet* were considered.

TABLE S4. | **Human ACE2 receptor (hACE2) *quartets* and their interaction energies [kcal/mol][a] with neighboring[b] virus S-RBD residues.**

Quartet	**Human ACE2 Receptor Residues**	E_{Int}^{DFT}	E_{Int}^{DD}	E_{Int}^{Total}
AQ1	ASP30-LYS31-ASN33-HIS34	+15.56	−12.78	+2.79
AQ2	GLN24-ALA25-LYS26-THR27	+50.14	−6.95	+43.19
AQ3	GLU329-ASN330-LYS353-GLY354	−38.93	−25.97	−64.91
AQ4	GLU37-ASP38-TYR41-GLN42	−46.12	−15.07	−61.20
AQ5	LEU91-THR92-GLN325-GLY326	+11.14	−2.24	+8.90
AQ6	MET82-TYR83-GLN89-ASN90	+32.51	−4.65	+27.86
AQ7	SER44-LEU45-ALA46-SER47	+27.18	−2.57	+24.61
AQ8	SER77-THR78-LEU79-ALA80	+26.06	−1.99	+24.06

[a] DFT energies computed at 6-311G(d,p)/B3LYP level; Dispersion (DD) corrections evaluated with semiempirical method. [2]

[b] All *concap* S-RBD residues within 4.5 Å of ACE2 *quartet* were considered.

TABLE S5. | **S-RBD-centered *quartets* and their interaction energies [kcal/mol][a] with neighboring[b] hACE2 residues.**

Quartet	SARS-CoV-1 S-RBD Residues	E_{Int}^{DFT}	E_{Int}^{DD}	E_{Int}^{Total}
SQ1	ASN435-TYR436-ASN437-TYR438	−12.81	−3.03	−15.83
SQ2	LYS439-TYR440-LEU478-ASN479	+50.88	−3.97	+46.90
SQ3	PHE483-TYR484-THR485-THR486	+25.18	−19.40	+5.78
SQ4	PRO470-ALA471-LEU472-ASN473	+19.92	−8.26	+11.66
SQ5	THR425-ARG426-ASN427-ILE428	−58.92	−2.09	−61.01
SQ6	THR487-GLY488-TYR491-GLN492	−19.40	−25.68	−45.08
SQ7	TYR442-LEU443-TYR475-TRP476	+16.16	−16.51	−0.35

[a] DFT energies computed at 6-31G*/B3LYP level; Dispersion (DD) corrections evaluated with semiempirical method. [2]
[b] All ACE2 residues within 4.5 Å of each S-RBD *quartet* were included.

TABLE S6. | **S-RBD-centered *quartets* and their interaction energies [kcal/mol][a] with neighboring[b] hACE2 residues.**

Quartet	SARS-CoV-1 S-RBD Residues	E_{Int}^{DFT}	E_{Int}^{DD}	E_{Int}^{Total}
SQ1	ASN435-TYR436-ASN437-TYR438	−10.14	−3.40	−13.54
SQ2	LYS439-TYR440-LEU478-ASN479	+51.89	−4.09	+47.80
SQ3	PHE483-TYR484-THR485-THR486	+30.93	−20.90	+10.03
SQ4	PRO470-ALA471-LEU472-ASN473	+24.10	−8.99	+15.11
SQ5	THR425-ARG426-ASN427-ILE428	−55.53	−2.32	−57.85
SQ6	THR487-GLY488-TYR491-GLN492	−14.18	−28.08	−42.27
SQ7	TYR442-LEU443-TYR475-TRP476	+17.85	−17.02	+0.83

[a] DFT energies computed at 6-31+G*/B3LYP level; Dispersion (DD) corrections evaluated with semiempirical method. [2]
[b] All ACE2 residues within 4.5 Å of each S-RBD *quartet* were included.

TABLE S7. | **S-RBD-centered *quartets* and their interaction energies [kcal/mol][a] with neighboring[b] hACE2 residues.**

Quartet	**SARS-CoV-1 S-RBD Residues**	E_{Int}^{DFT}	E_{Int}^{DD}	E_{Int}^{Total}
SQ1	ASN435-TYR436-ASN437-TYR438	-12.49	-3.03	-15.51
SQ2	LYS439-TYR440-LEU478-ASN479	$+51.17$	-3.97	$+47.20$
SQ3	PHE483-TYR484-THR485-THR486	$+26.27$	-19.40	$+6.88$
SQ4	PRO470-ALA471-LEU472-ASN473	$+22.08$	-8.26	$+13.82$
SQ5	THR425-ARG426-ASN427-ILE428	-57.96	-2.09	-60.05
SQ6	THR487-GLY488-TYR491-GLN492	-17.87	-25.68	-43.55
SQ7	TYR442-LEU443-TYR475-TRP476	$+17.19$	-16.51	$+0.68$

[a] DFT energies computed at 6-311G(d,p)/B3LYP level; Dispersion (DD) corrections evaluated with semiempirical method. [2]

[b] All ACE2 residues within 4.5 Å of each S-RBD *quartet* were included.

REFERENCES

[1] Becke, A. D. A new mixing of Hartree–Fock and local density-functional theories. *J. Chem. Phys.* **98**, 1372–1377 (1993).

[2] Deligkaris, C. & Rodriguez, J. H. Correction to DFT interaction energies by an empirical dispersion term valid for a range of intermolecular distances. *Phys. Chem. Chem. Phys.* **14**, 3414–3424 (2012).

[3] Cossi, M., Rega, N., Scalmani, G. & Barone, V. Energies, structures, and electronic properties of molecules in solution with the C-PCM solvation model. *J. Comp. Chem.* **24**, 669–681 (2003).

[4] Marenich, A. V., Cramer, C. J. & Truhlar, D. G. Universal solvation model based on solute electron density and on a continuum model of the solvent defined by the bulk dielectric constant and atomic surface tensions. *J. Phys. Chem. B* **113**, 6378–6396 (2009).
